# Does increased implementation support improve community clinics’ guideline-concordant care? Results of a mixed methods, pragmatic comparative effectiveness trial

**DOI:** 10.1186/s13012-019-0948-5

**Published:** 2019-12-05

**Authors:** Rachel Gold, Arwen Bunce, Stuart Cowburn, James V. Davis, Joan C. Nelson, Christine A. Nelson, Elisabeth Hicks, Deborah J. Cohen, Michael A. Horberg, Gerardo Melgar, James W. Dearing, Janet Seabrook, Ned Mossman, Joanna Bulkley

**Affiliations:** 1 Kaiser Permanente Center for Health Research, 3800 N Interstate Ave, Portland, OR 97227 USA; 2 OCHIN, Inc., 1881 NW Naito Pkwy, Portland, OR 97201 USA; 3 Oregon Health & Science University, 3181 SW Sam Jackson Park Rd, Portland, OR 97239 USA; 4 Kaiser Permanente Mid-Atlantic Permanente Research Institute, 2101 East Jefferson St, Rockville, MD 20852 USA; 5 Cowlitz Family Health Center, 1057 12th Avenue, Longview, WA 98632 USA; 6 Michigan State University, 404 Wilson Rd, Room 473, East Lansing, MI 48824 USA; 7 Community HealthNet Health Centers, 1021 West 5th Avenue, Gary, IN 46402 USA

**Keywords:** Implementation support, Community health centers, Guideline-concordant care

## Abstract

**Background:**

Disseminating care guidelines into clinical practice remains challenging, partly due to inadequate evidence on how best to help clinics incorporate new guidelines into routine care. This is particularly true in safety net community health centers (CHCs).

**Methods:**

This pragmatic comparative effectiveness trial used a parallel mixed methods design. Twenty-nine CHC clinics were randomized to receive increasingly intensive implementation support (implementation toolkit (arm 1); toolkit + in-person training + training webinars (arm 2); toolkit + training + webinars + offered practice facilitation (arm 3)) targeting uptake of electronic health record (EHR) tools focused on guideline-concordant cardioprotective prescribing for patients with diabetes. Outcomes were compared across study arms, to test whether increased support yielded additive improvements, and with 137 non-study CHCs that share the same EHR as the study clinics. Quantitative data from the CHCs’ EHR were used to compare the magnitude of change in guideline-concordant ACE/ARB and statin prescribing, using adjusted Poisson regressions. Qualitative data collected using diverse methods (e.g., interviews, observations) identified factors influencing the quantitative outcomes.

**Results:**

Outcomes at CHCs receiving higher-intensity support did not improve in an additive pattern. ACE/ARB prescribing did not improve in any CHC group. Statin prescribing improved overall and was significantly greater only in the arm 1 and arm 2 CHCs compared with the non-study CHCs. Factors influencing the finding of no additive impact included: aspects of the EHR tools that reduced their utility, barriers to providing the intended implementation support, and study design elements, e.g., inability to adapt the provided support. Factors influencing overall improvements in statin outcomes likely included a secular trend in awareness of statin prescribing guidelines, selection bias where motivated clinics volunteered for the study, and study participation focusing clinic staff on the targeted outcomes.

****Conclusions**:**

Efforts to implement care guidelines should: ensure adaptability when providing implementation support and conduct formative evaluations to determine the optimal form of such support for a given clinic; consider how study data collection influences adoption; and consider barriers to clinics’ ability to use/accept implementation support as planned. More research is needed on supporting change implementation in under-resourced settings like CHCs.

**Trial registration:**

ClinicalTrials.gov, NCT02325531. Registered 15 December 2014.

Contributions to the literature

- Little is known about the comparative effectiveness of commonly -used implementation strategies, or whether increased amounts of implementation support yield increased adoption of innovations, especially in safety net community health centers. This study adds evidence on implementation strategies used to support adoption of guideline-based clinical decision support tools in this important healthcare setting.
- Our understanding of the challenges inherent to providing implementation support is nascent; this study illustrates the multi-level challenges that may be faced in providing implementation support as planned, showing that there are barriers to providing such support with fidelity, just as to providing interventions with fidelity.

## Background

Disseminating adoption of evidence-based care guidelines into widespread clinical practice remains challenging [1]. One reason is a lack of evidence about how best to support clinics as they implement new guidelines into routine care; this is particularly true for community health centers (CHCs) serving socioeconomically vulnerable patients [2]. Evidence shows that clinics usually need support (called implementation strategies) [3, 4] in changing care patterns. Past research has assessed use of implementation strategies (e.g*.*, education, facilitation, audit and feedback) to help clinics adopt a given intervention [5–12], but few studies have directly compared which implementation strategy or combination of strategies most effectively support implementing guidelines or other innovations in CHCs or in any care setting [13–17].

This is also true in our research. In a previous study, we helped 11 CHC clinics adopt electronic health record (EHR)-based clinical decision support (CDS) tools that targeted guideline-concordant prescribing in patients with diabetes mellitus (DM) [7]. The implementation strategies we provided involved peer-led training/facilitation from study-funded clinic staff, monthly meetings to engage and support clinic champions, and regular audit and feedback provided by the study team [3, 4]. This support was associated with a 38% relative improvement in targeted outcomes in the intervention clinics, versus no change in the control clinics.

However, these implementation strategies were costly, leading us to question whether the same improvements could be achieved with less intensive support. Thus, we conducted the Study of Practices Enabling Implementation and Adaptation in the Safety Net (SPREAD-NET) to assess whether more scalable implementation strategies support adoption of cardioprotective prescribing guidelines in CHCs (our main study objective). We compared the effectiveness of increasingly intensive implementation support and explored the factors impacting this effectiveness. Our primary hypothesis was that increased implementation support would be associated with increased improvements in prescribing rates of cardioprotective medications (ACE/ARBs and statins), with an additive effect. Our secondary quantitative hypotheses were that accurate (per current guidelines) statin dosage would improve in a similarly additive manner, and that any level of support would yield better outcomes than no support. Qualitative data collection was purposefully not hypothesis-driven; rather, we sought to gain a context-specific understanding of the implementation process at each site to inform understanding of the (as yet unknown) intervention outcomes, and to increase the credibility and transferability of study findings [18]. This is one of the first studies [19] to directly assess increasingly intensive implementation support in CHCs [20, 21].

## Methods

### Overview

The planned methods of this mixed methods, pragmatic comparative effectiveness trial were reported previously [22]. In brief, an earlier version of the innovation (EHR-embedded CDS tools) targeted here was shown to be effective and feasible to implement in CHCs, in our previous trial. Prior to the current trial, the EHR provider adapted and expanded the scope of these tools; the modified innovation is called the cardiovascular disease (CVD) bundle. In the current pragmatic trial, 29 CHCs were randomized to be offered increasingly intensive implementation support designed to enhance implementation of this innovation. Randomization was conducted by the study team’s statisticians. They calculated that 29 clinics would provide adequate power to detect differences in changes between study arms of >=8%, with power of .95–.99 at an intra-class correlation of .01, and power of .76 to .99 if .02, with an alpha level of .05. The Practice Change Model, which identifies factors specific to primary care practice change that can influence intervention uptake, was the conceptual model underlying this study [23, 24].

### Setting

All study CHCs were members of OCHIN, Inc. (not an acronym), a non-profit organization based in Portland, OR, that provides health information technology to > 600 CHC clinics around the U.S., including a shared Epic© ambulatory EHR. Any OCHIN member clinics that provide primary care to adult patients were considered eligible to participate; recruitment involved OCHIN staff reaching out to clinic leadership to assess their interest.

The 29 OCHIN member clinics recruited to the study were managed by 12 CHC organizations in six states. They were cluster-randomized to receive low (arm 1, *n* = 9), medium (arm 2, *n* = 11), or high-intensity (arm 3, *n* = 9) implementation support (details below) targeting adoption of the CVD bundle. Randomization was by organization, weighted based on number of patients with DM, number of clinics, and urban/rural location. (During the study period, one organization closed, so two arm 3 clinics were lost to follow-up after October 2016; another organization left OCHIN, so two arm 2 clinics were lost to follow-up after October 2017. Data from these sites were truncated in all analyses.) Since the innovation was also available to all of the non-study CHCs that were OCHIN members during the study period, we identified a set of similar clinics (*n* = 137) as a natural comparison group for the use in quantitative analyses.

### The innovation: the CDS “CVD bundle”

In our previous study, the EHR tools included point-of-care alerts that appeared when a patient with DM was indicated for but not currently prescribed an ACE/ARB and/or a statin, order sets to expedite prescribing these medications, and data rosters that identified all patients in a given population who lacked an indicated prescription. As noted above, prior to this study, these tools were adapted to incorporate new statin prescribing guidelines, including appropriate dosage. In addition, the CVD bundle included panel management data tools that could be used to identify patients indicated for but not prescribed an ACE/ARB or statin, and to track clinic progress in changing these prescribing patterns. There were also alerts and roster tools targeting other aspects of DM care, including alerts to promote accurate charting. This suite of tools was considerably more complex than that tested in our prior study.

### Timeline (see Fig. 1)

To capture guideline-concordant prescribing patterns over the study period, we evaluated quantitative data covering 48 months (May 2014 to April 2018), conceptualized as follows: pre-intervention (May 2014–June 2015; months 1–14), intervention (July 2015–June 2016; months 15–26), and maintenance (July 2016–April 2018; months 27–48). (Though some intervention components occurred in June 2015, July 2015 was the first full month of the intervention period. Additionally, elements of the arms 2–3 intervention extended into the first year of the maintenance period (Table 1). During this period, the comparison clinics received no implementation support.)

**Fig. 1:**
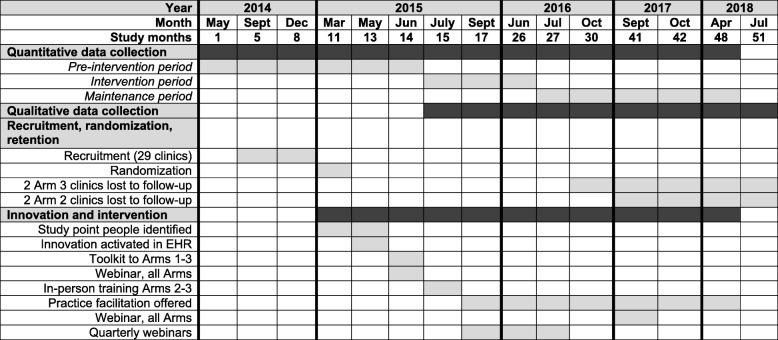
Study flow timeline

**Table 1:** Implementation support strategies provided to each study arm’s CHCs

Implementation support strategies	ERIC category [3, 4]	Arm 1	Arm 2	Arm 3
Spring 2015: Identify a study “Point Person” and/or a “Clinician Champion” [21]. Diverse staff filled these roles.	Identify and prepare champions	X	X	X
June 2015: Clinics receive “CVD bundle implementation toolkit’ on how to find/use the CVD bundle components, and practice change techniques.	Develop/distribute educational materials	X	X	X
June 2015: Webinar on the CVD bundle.	Conduct ongoing training	X	X	X
June 2016: Webinar on minor changes made to the CVD bundle and the toolkit.	Conduct ongoing training	X	X	X
July 2015: 2–3 clinic representatives attend 2-day in-person training, all costs covered. Training covered lessons from our prior trial, hands-on practice using the CVD bundle and implementation toolkit, change management techniques.	Conduct ongoing training/make training dynamic		X	X
September 2015-August 2016: Quarterly webinars with content based on identified training needs.	Conduct ongoing training		X	X
September 2015–April 2018: Offered practice facilitation > = 2 onsite visits in the first year; > = 3 visits over the 2nd and 3rd years. Purpose was to help address barriers to implementing the CVD bundle. PF visits included meetings with point people, clinician champions, and clinic leadership, interviews with clinic employees to understand clinic functioning and capacity, and training on the guidelines underlying the CVD bundle. At initial visit, PF also spoke at staff/provider meetings. After that visit, PF provided virtual coaching tailored to each organization’s needs, including monthly emails with study point people, webinars for clinic staff, and connecting staff to other resources (e.g., technical support). A second visit occurred at all arm 3 CHCs in March–May 2016; these visits’ content varied in response to clinic needs.	Facilitation			X

### The intervention: implementation support

The support offered to the study arms’ CHCs was comprised of combinations of implementation strategies. These strategies, chosen for their scalability and demonstrated ability to support practice change in certain settings [3, 25–33], are summarized in Table 1.

### Study data

In this convergent parallelmixed-methods design [34], quantitative and qualitative data were collected and analyzed concurrently but separately, and the two sets of complementary results merged in study year 5, during the interpretive phase, to develop a more comprehensive understanding of the process under study. This was done as a partnership between the study’s qualitative and quantitative teams. In this way, we quantitatively measured the impact of each implementation support approach on prescribing rates, then used qualitative findings to understand the factors associated with the quantitative results. All EHR-based quantitative data were extracted from OCHIN’s database using structured SQL queries.

Clinic-reported baseline data collection prior to the intervention was overseen by each clinic’s study Point Person, including an all-staff survey on clinic context, perceived quality improvement needs, and staff demographics, and another survey completed by one person (e.g*.*, clinic manager) at each clinic, covering the clinic’s ownership structure, staffing, revenue, billing, and insurance characteristics, and experiences with implementing practice change [35–37].

Qualitative data on clinic experiences with the CVD bundle and the offered implementation support were collected via multiple modalities in real time throughout the intervention and maintenance periods. This prolonged engagement and triangulation of methods and sources [38] facilitated a deeper understanding of clinic implementation activities, barriers, and facilitators from multiple perspectives. Study staff called clinic Point Persons twice monthly in study months 16–21 (assumed to be a period of intense implementation activity), and once monthly in study months 22–33, to learn about their clinic’s progress in adopting and using the CVD bundle. After this point, since implementation efforts had plateaued, call frequency was further reduced. At these calls, most of which lasted a minimum of 10 minutes, the point people were asked about their clinic’s progress in adopting the CVD bundle, whether/how the provided implementation support facilitated this adoption, and what factors might be impacting response to and use of the CVD bundle; we also checked our evolving understanding of site-specific implementation processes and determinants. In addition, we conducted 2-day site visits at eight of the 12 study organizations. At these visits, we shadowed care teams caring for patients with DM, to observe each step of the encounter, focusing on EHR use and discussion of medications; observed relevant team and clinic meetings, including huddles and quality improvement discussions; and interviewed 6–21 providers and staff per organization for approximately 20 minutes each about their approach to prescribing ACE/ARBs and statins to patients with DM, and use of the CVD bundle, as well as organizational approaches to care standardization. Interviewees were purposefully selected to maximize variation in experience with and perspectives on the CVD bundle and the implementation process; sample size was dictated by clinic size and staff availability. We also debriefed with the study practice facilitator following her arm 3 site visits, and we reviewed her field notes, to understand the impact of these visits on adoption of the CVD bundle, and to gain additional insight into implementation progress at these clinics. All calls and interviews were recorded and transcribed for analysis.

### **Analyses**

#### Quantitative

Our primary outcome was proportion of patients in a given arm’s CHCs who had DM and were indicated for cardioprotective medication(s) (denominator) and had a prescription for a given medication(s) (numerator), calculated monthly for statins and ACE/ARBs. Each monthly denominator included patients who had an in-clinic encounter in the last year and were indicated for the medication per national guidelines. Pregnant/breastfeeding patients were excluded; patients with a history of anaphylactic reaction to either medication were excluded from analyses involving that medication. Patients were considered to have a prescription for a medication if it was prescribed in the previous year, to reflect prescription data available in the EHR. Our secondary analyses assessed change in proportion of patients prescribed the correct statin dosage per current guidelines.

We used a difference-in-difference (DiD) [39] approach to evaluate the pre/post change in prescribing rate(s) within each study arm and relative to the comparison clinics. DiD models utilized generalized estimating equation (GEE) Poisson regression with a robust error variance [40] to calculate rate ratios (RRs) with 95% confidence intervals (CIs). To account for potential differences in prescribing rates arising from dissimilarities in patient-, provider-, and encounter-level factors across arms, we adjusted all models for time-invariant (patient sex, race/ethnicity, preferred language) and time-varying covariates (age, household federal poverty level [FPL] at last visit, insurance status at last visit, any office visit in the last 6 months, number of office visits in the last year, whether the primary care provider [PCP] was seen at most recent visit, PCP type [MD/DO/Resident, NP/PA, other, unknown], last HbA1c, last LDL, last blood pressure, last body mass index, last smoking status, adapted Charlson comorbidity index, and comorbidities in addition to DM (yes/no)). All covariates were treated as categorical. All analyses reflect tests of statistical significance with a two-sided *α* of 0.05 and were conducted using SAS Enterprise Guide 7.15 (SAS Institute Inc., Cary, NC, USA).

#### Qualitative

While our quantitative analyses tested our hypothesis that increased implementation support would be associated with similarly increased rates of guideline-concordant cardioprotective prescribing, our qualitative analyses aimed to explain the interconnected factors affecting how the offered implementation support impacted the study clinics. Coding and preliminary analyses were blinded to quantitative study outcomes. Quantitative and qualitative results were merged in year 5, and the qualitative findings used to inform our understanding of the “how” and “why” behind the observed outcomes.

Starting in the maintenance period, three qualitative researchers separately read and made notes about data gathered to that point. The lead qualitative researcher developed an initial code list and definitions, which were then collaboratively reviewed and revised to create a preliminary codebook [41]. The codes were then applied to the same sample of transcripts by all three members of the qualitative team and iteratively revised as indicated. Once the codes and definitions were solidified and applied consistently across all coders, each qualitative researcher was given data to code independently. Coding was conducted in the QSR NVivo software, guided by the constant comparative method [42, 43]. As additional data were collected and analyzed, codes and definitions were revised as necessary. Five percent of all qualitative data was double-coded to ensure consistent coding; inconsistencies were resolved through team discussion. When applicable, results are presented along with the Consolidated Framework for Implementation Research (CFIR) categories with which they align, to enable their comparison to those of similar studies [44, 45].

## Results

At the start of the pre-intervention period (May 2014), a majority of the study arms’ CHCs patient panels were age 40–75, English-speaking, and female (Table 2). Differences in the arms’ CHCs’ patients’ demographic characteristics (distribution by race/ethnicity, language, income, and insurance coverage) were generally more pronounced than those in clinical characteristics (LDL control, HbA1c control, blood pressure control). Eighty-six percent (*N* = 3,299) of the arm 1 clinics’ patients with DM were indicated for statins, as were 83% (*N* = 4,239) in the arm 2 clinics, 85% (*N* = 2,850) in the arm 3 clinics, and 84% (*N* = 28,257) in the comparison clinics. The proportion of each arm’s clinics’ patients indicated for ACE/ARBs was 67%, 70%, 70%, and 68% for arms 1, 2, 3, and the comparison CHCs, respectively.

**Table 2:** Characteristics of study CHCs’ patients with diabetes as of May 30, 2014 (beginning of pre-intervention period)

	Arm 1 CHCs		Arm 2 CHCs		Arm 3 CHCs		Comparison CHCs		*p* value
	*N*	(%)	*N*	(%)	*N*	(%)	*N*	(%)	
Total patients	3849		5098		3370		33,638		
Indicated for statin use	3299	(85.7)	4239	(83.2)	2850	(84.6)	28,257	(84.0)	0.009
Indicated for ACE/ARB use	2589	(67.3)	3580	(70.2)	2369	(70.3)	22,808	(67.8)	< 0.001
Age (years)									0.009
18–21	31	(0.8)	33	(0.6)	24	(0.7)	212	(0.6)	
22 to 39	357	(9.3)	574	(11.3)	337	(10.0)	3649	(10.8)	
40 to 75	3169	(82.3)	4064	(79.7)	2744	(81.4)	27,309	(81.2)	
> 75	292	(7.6)	427	(8.4)	265	(7.9)	2468	(7.3)	
Gender									< 0.001
Female	2047	(53.2)	3057	(60.0)	1831	(54.3)	18,707	(55.6)	
Male	1802	(46.8)	2041	(40.0)	1539	(45.7)	14,931	(44.4)	
Race/ethnicity									< 0.001
Non-Hispanic White	2,417	(62.8)	880	(17.3)	2389	(70.9)	15,582	(46.3)	
Non-Hispanic Black	44	(1.1)	1364	(26.8)	196	(5.8)	7056	(21.0)	
Non-Hispanic Other	127	(3.3)	350	(6.9)	126	(3.7)	2134	(6.3)	
Hispanic	1249	(32.4)	2494	(48.9)	652	(19.3)	8734	(26.0)	
Unknown	12	(0.3)	10	(0.2)	7	(0.2)	132	(0.4)	
Patient preferred language									< 0.001
English	2781	(72.3)	3259	(63.9)	2883	(85.5)	23,979	(71.3)	
Spanish	993	(25.8)	1690	(33.2)	395	(11.7)	7011	(20.8)	
Other	46	(1.2)	138	(2.7)	79	(2.3)	2372	(7.1)	
Unknown	29	(0.8)	11	(0.2)	13	(0.4)	276	(0.8)	
Household income as % of FPL*									< 0.001
0 to 100%	1829	(47.5)	1456	(28.6)	1317	(39.1)	17,999	(53.5)	
101 to 200%	776	(20.2)	425	(8.3)	758	(22.5)	6393	(19.0)	
> 200%	122	(3.2)	62	(1.2)	287	(8.5)	2271	(6.8)	
Unknown	1122	(29.2)	3155	(61.9)	1008	(29.9)	6975	(20.7)	
Insurance status*									< 0.001
Medicaid	1421	(36.9)	1924	(37.7)	1089	(32.3)	11,436	(34.0)	
Medicare	1403	(36.5)	1725	(33.8)	1315	(39.0)	10,386	(30.9)	
Other public	41	(1.1)	8	(0.2)	23	(0.7)	742	(2.2)	
Private	397	(10.3)	400	(7.8)	504	(15.0)	3829	(11.4)	
Uninsured	587	(15.3)	1041	(20.4)	439	(13.0)	7245	(21.5)	
LDL control (mg/dL)**									< 0.001
Not in control (> 129 mg/dL)	512	(13.3)	733	(14.4)	482	(14.3)	5311	(15.8)	
In control (< = 129 mg/dL)	2643	(68.7)	3453	(67.7)	2373	(70.4)	22,888	(68.0)	
Unknown	694	(18.0)	912	(17.9)	515	(15.3)	5439	(16.2)	
HbA1c control (%)**									< 0.001
Not in control (> = 7)	2022	(52.5)	2547	(50.0)	1735	(51.5)	17,265	(51.3)	
In control (< 7)	1561	(40.6)	2020	(39.6)	1428	(42.4)	14,186	(42.2)	
Unknown	266	(6.9)	531	(10.4)	207	(6.1)	2187	(6.5)	
Blood pressure control (mm Hg)**									< 0.001
Not in control	910	(23.6)	1,174	(23.0)	702	(20.8)	8258	(24.5)	
In control (< 140/90:< 60 yo, < 150/90:> = 60 yo)	2939	(76.4)	3923	(77.0)	2668	(79.2)	25,355	(75.4)	
Unknown	-	*-*	1	(0.0)	-	*-*	25	(0.1)	

*Assessed at most recent office visit prior to snapshot date **Most recent record available prior to snapshot date. P values from chi-square test for general association

***ACE/ARB*** angiotensin-converting-enzyme inhibitor/Angiotensin II receptor blockers, CHC community health center, FPL Federal Poverty Level, HbA1c Hemoglobin A1c, LDL Low-density lipoprotein

**Fig. 2:**
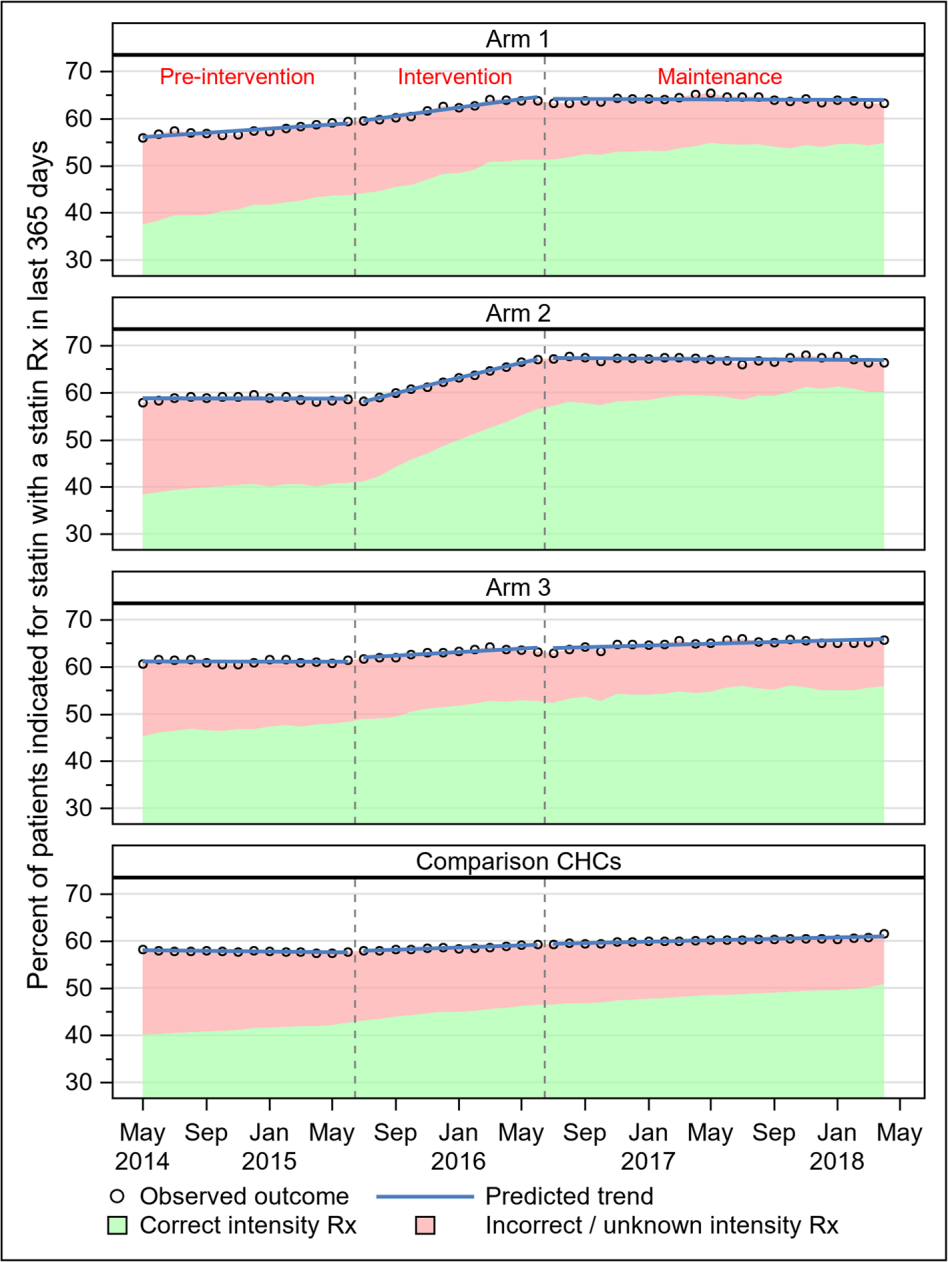
Statin prescribing by month and study arm

In sum, CHCs that received higher intensity implementation support did not show greater improvement in prescribing in an additive manner, although some improvements in statin prescribing were seen. Three general patterns were observed in statin prescribing over the study period (Fig. 2). In the first pattern (observed in arm 1), prescribing rates increased in a roughly linear fashion over both the pre-intervention and intervention periods, then plateaued in the maintenance period. In the second pattern (observed in arm 2), prescribing rates were flat in the pre-intervention period, increased during the intervention period, then plateaued in the maintenance period. In the third pattern (observed in both arm 3 and the comparison CHCs), monthly statin prescribing rates remained flat during the pre-intervention period, then increased very modestly in a roughly linear fashion across both the intervention and maintenance periods. In the adjusted DiD model, arms 1 and 2 experienced significantly greater increases in statin prescribing over the study period than the comparison CHCs did: 5% greater in arm 1 (RR 1.05; 95% CI 1.01–1.08), and 6% greater in arm 2 (RR 1.06; 95% CI 1.03–1.09) (Table 3). Prescribing gains in arm 3 were no different from those in the comparison CHCs (RR 1.02; 95% CI 0.99–1.06).

**Table 3:** Results from difference-in-difference models estimating changes in prescribing rate(s) from pre-implementation to maintenance periods

Outcome	Comparison	Arm 1 RR (95% CI)	Arm 2 RR (95% CI)	Arm 3 RR (95% CI)	Comparison CHCs RR (95% CI)
Primary outcomes					
Statin prescribing	Change within arm	*1.09 (1.06–1.13)*	*1.11 (1.08–1.13)*	*1.06 (1.03–1.09)*	*1.04 (1.03–1.05)*
	Change relative to comparison CHCs	*1.05 (1.01–1.08)*	*1.06 (1.03–1.09)*	1.02 (0.99–1.06)	-
ACE/ARB prescribing	Change within arm	*0.95 (0.92–0.97)*	0.99 (0.97–1.01)	0.99 (0.96–1.02)	*0.95 (0.94–0.95)*
	Change relative to comparison CHCs	1.00 (0.98–1.03)	*1.05 (1.02–1.08)*	*1.05 (1.02–1.08)*	-
Secondary outcomes					
Correct intensity statin prescribing	Change within arm	*1.24 (1.21–1.28)*	*1.35 (1.31–1.39)*	*1.11 (1.08–1.15)*	*1.17 (1.16–1.18)*
	Change relative to comparison CHCs	*1.06 (1.03–1.10)*	*1.15 (1.12–1.19)*	*0.95 (0.93–0.98)*	*-*

*RR* adjusted Rate Ratio, *CI* confidence interval. Results in italics are statistically significant at *α* = 0.05

### Statin prescribing

Statin dosage prescribing patterns were similar to those for statin prescribing in general, except that correct intensity prescribing improved faster (i.e., slopes of trends within each time period were steeper) than those in statin prescribing overall (Fig. 2). Over the entire study period, arms 1 and 2 demonstrated larger increases in correct intensity prescribing than the comparison CHCs (arm 1 RR 1.06; 95% CI 1.03–1.10; arm 2 RR 1.15; 95% CI 1.12–1.19) (Table 3); arm 3’s increase in correct-intensity prescribing was significantly smaller than that in the comparison CHCs (RR 0.95; 95% CI 0.93–0.98).

### ACE/ARB prescribing

**Fig. 3:**
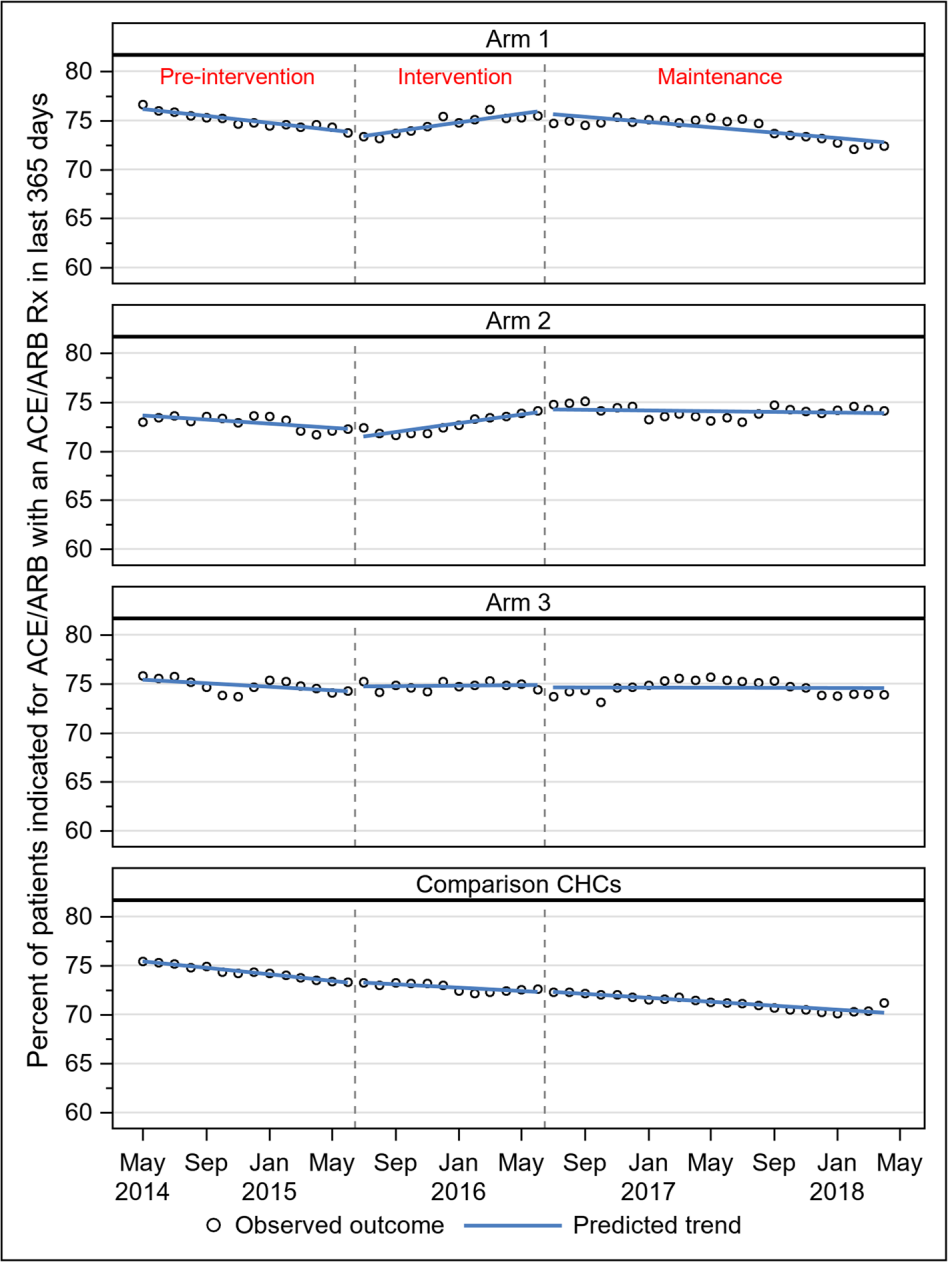
ACE/ARB prescribing by month and study arm

As with statin prescribing, three patterns in ACE/ARB prescribing were observed (Fig. 3). In arms 1 and 2, prescribing declined slightly during the pre-implementation period, increased modestly during the implementation period, then declined again during the maintenance period. Prescribing rates in arm 3 were essentially flat across all observed periods. In the comparison CHCs, monthly rates declined in roughly linear fashion across all observed periods. In DiD models, the pre/post change in ACE/ARB prescribing from pre-intervention to maintenance periods in arm 1 was not significantly different from the equivalent in the non-study CHCs (RR 1.00; 95% CI 0.98–1.03) (Table 3). Both arms 2 and 3 experienced a relative improvement in ACE/ARB prescribing compared with the non-study CHCs (arm 2 RR 1.05; 95% CI 1.02–1.08; arm 3 RR 1.05; 95% CI 1.02–1.08).

### Factors impacting prescribing outcomes

Qualitative analysis identified an interconnected set of factors that impacted the results described above.

#### Problems with the innovation

First, attributes of the innovation negatively affected its implementation. The CVD bundle was not perfectly compatible with practice requirements, as some of its content did not align with quality measures that the study CHCs had to address in value-based payment structures: i.e., the CVD bundle highlighted cardioprotective prescribing, while the clinics’ quality measures focused on biomarker control (CFIR: intervention—design quality). For the first two years after the CVD bundle went live, the host EHR did not include a 10-year CV risk calculator; as a result, some of the Bundle’s statin-related alerts simply referred users to use a web-based calculator (CFIR: intervention–complexity). (When the risk calculator was added to the EHR, the alerts were amended to draw on its results.) The CVD bundle’s tools were not optimized for team-based care: their override function was specific to individual staff, so a provider might override an alert for a given patient, but the other care team members or PCPs would still see the alert; and the alerts could only be accessed in an open encounter, making them less useful for chart review or pre-visit planning/“scrubbing” (CFIR: intervention–design quality). In addition, the roster tools proved very difficult to use, limiting clinics’ ability to track their own progress (CFIR: intervention–complexity). In some cases, the tools’ accuracy was questioned when clinic staff’s variable knowledge about the underlying care guidelines affected perceptions of the tools’ accuracy (CFIR: intervention–evidence). Given these issues, some clinic leadership or staff felt the tools were not worth promoting.

#### Providing implementation support as planned

Second, we encountered barriers to providing the implementation support strategies as intended. All study clinics were mailed the “CVD Bundle Implementation Toolkit”, and an electronic version was emailed to the study Point Person (Appendix 1). The toolkit was designed to be modular, but some study clinic staff found it overly complex; those that used it generally considered the EHR “how-to” sections most useful (CFIR: intervention–complexity, here applied to the implementation support rather than the intervention).

Staff from all 12 study organizations in the study attended the first training webinar in April 2015, shortly before the “CVD bundle” went live (1–5 people per organization), Table 4. Though all subsequent webinars were tailored to address training needs identified from qualitative data, webinar attendance was inconsistent (Table 5 shows webinar dates and topics). Attendee discussion and sharing of learning were encouraged at each webinar, but rarely occurred (CFIR: process–engaging). Due to the low levels of engagement, the study team reduced the overall number of webinars provided and offered them only when a training need was clearly identified.

**Table 4:** Attendance at trainings

Organization	# Clinics	Arm	Orientation WebEx attendees (#)	In-person training attendees (#)	Trainee clinic roles
1	1	1	1	-	-
2	5	1	5	-	-
3	1	1	2	-	-
4	2	1	1	-	-
5	2	2	2	1	MD
6	5	2	1	3	Medical director, 2 IT/EHR staff
7	2	2	1	3	MD, MA, NP
8	2	2	3	1	MD
9	4	3	4	4	Patient advocacy manager; site manager; QI specialist; office supervisor
10	1	3	1	1	Clinical data analyst
11*	2	3	2	2	Medical director, RN/QA coordinator
12	2	3	3	3	Pharmacist, pharmacy director, NP

*This organization closed in June 2016 so was lost to further follow-up

**Table 5:** **Webinars**

Date	Topic	Invited arms
September 26, 2015	Troubleshooting issues with the CVD risk management bundle	2–3
December 10, 2015	Alerts, tools, reporting and documentation issues with the CVD risk management bundle	2–3
March 7, 2016	Summary of the clinical guidelines behind the CVD bundle	2–3
March 8, 2016	How to run reports in reporting workbench: a real time demonstration	2–3
May 19, 2016	Spread-net annual Webinar: updates to the CVD bundle and toolkit	1–3
August 9, 2016	OCHIN’s “Diabetes Improvement Guide” webinar	1–3

Eighteen staff from arm 2–3 clinics attended the July 2015 in-person training, but remote training was necessary for two staff members unable to attend the arm 2–3 in-person training. Several of the Point Person trainees left their jobs during the study period, and we had limited ability to train their replacements (CFIR: process–executing).

Though the support of a practice facilitator (PF) was offered to the arm 3 CHCs, all of which had at least one PF visit and subsequent interactions with the PF (Table 1), none of the arm 3 sites took advantage of all of the additional PF visits offered by the study, so they did not receive the full PF dose as planned (CFIR: process–executing; engaging). Thus, while the study was designed to let clinic staff tell the PF how to help them enhance their adoption of the CVD bundle, doing so proved challenging. As a result, the provided facilitation often focused on support for overall DM care management processes.

#### The study design

Third, elements of the study design had unintended impacts on the study results. The clinics’ study point people had variable influence and authority at their clinic, and variable clinical/quality improvement skills; the training webinars could not be tailored to meet all attendees’ needs; no follow-up on the in-person training for arms 2–3 was feasible, even when the point people who had been trained were replaced by others who had not; and the provided implementation support could not be customized to a given clinic’s needs (CFIR: intervention–adaptability; inner setting–structural characteristics). Further, ongoing interactions with the study team may have focused some clinic staff’s attention on the targeted outcomes. For example, a few staff reported that their qualitative team check-ins kept the targeted outcomes on their radar. Further, it appeared—based on the calls’ timing—that the qualitative team’s calls and site visits spurred some provider and/or clinic-wide conversations related to the relevant guidelines and CDS tools.

### Differences by medication type

Qualitative findings also explain the differing results by medication type. Clinic staff generally did not focus on improving ACE/ARB prescribing rates: the ACE/ARB guidelines were perceived as relatively stable before and during the study period (apart from debates around appropriate blood pressure targets); in addition, ACE/ARB prescribing rates at study start were considerably higher than those for statins. Many of the study clinics did emphasize improving statin prescribing, especially in the intervention year, at least in part because the recent statin guideline changes were perceived to be substantial. Some of the study clinics took steps to improve statin prescribing; these actions, which help illuminate the related improvements seen here, included taping statin dosing tables to staff computers, providing links to the AHA/ACC risk calculator (before its addition to the CVD bundle), sharing provider-specific quality metrics, peer-to-peer discussions, engaging with clinical pharmacists, and leading by example. Notably, these efforts generally did not directly involve the CVD bundle. Augmenting these actions, many of the arm 2 clinics had highly engaged clinician champions that visibly supported these efforts.

## Discussion

The guideline-concordant cardioprotective prescribing targeted by the CVD bundle tools did not improve in an additive pattern as CHCs received increasing amounts of support to implement use of the tools; thus, our primary hypothesis was not upheld. This outcome was influenced by staff reactions to attributes of the innovation itself, problems with the targeted innovation, challenges involved in providing the implementation support as intended, and aspects of the study design. These findings have implications for understanding the effectiveness of implementation support strategies and how to study this effectiveness.

### Implementation strategies

In past studies, implementation strategies like those used here yielded improved outcomes, but only in some situations [3, 31–33, 46–48]: implementation toolkits (on their own or with other strategies) had mixed impact on provider behaviors [25, 27, 49, 50]; small-group in-person interactive trainings impacted provider performance [3, 26, 51–53]; multi-component training improved guideline-concordant prescribing in some contexts [25, 54, 55]; practice facilitation improved care quality in some cases [5, 9, 29, 30, 32, 56, 57], as did providing feedback data [31, 32, 58–62]. Here, the implementation toolkit was considered overwhelming, illustrating the challenge of providing adequate versus too much information in such guides; research is needed on whether and how toolkits can be optimized to better support practice change, or whether a different approach is needed [63]. Webinar attendance was not strong: for some clinic staff, even 30-minute webinars may be burdensome; and while attractively scalable, webinars may be too passive to engage or impact adult learners. Enthusiasm for the in-person training was high, but several point people left their clinics soon after the training; turnover is a known barrier to implementation success and sustainability. Further, the study point person who took part in the training had variable influence at their clinics, and variable EHR, clinical, and/or quality improvement expertise; research is needed on optimizing training approaches to support implementation, including assessing which trainees are likely to share learnings and be able to advocate for practice change post-training. It was difficult to deliver content in the webinars that was helpful to attendees with such variable competencies.

Our experience in providing PF is noteworthy. Our team’s PF was not an EHR trainer, but the clinics were interested in EHR optimization support; this suggests that if a practice change involves technological tools, the facilitator needs relevant training, or support from an EHR trainer. The PF visits did incur some successes: the PF provided training on quality improvement, EHR use, and the targeted care guidelines, as feasible. Though the PF adapted her approach as possible, clinic staff still had limited ability and time with which to capitalize on this offered resource. As a result, we could not provide PF with the planned intensity. These findings are similar to others’; for example, Seers et al. [21] found no differences in outcomes by facilitation “dose”, experienced challenges to providing facilitation as planned, and concluded that tailoring facilitation approaches to clinic context was essential. Rycroft-Malone et al. [20] found that facilitation’s success depended on whether the study sites prioritized the outcomes targeted by the facilitation.

### Study design

Aspects of the study design also impacted these results. We sought to compare the effectiveness of specific combinations of implementation strategies, so were unable to customize implementation support to each clinic’s specific needs. Recent evidence shows the importance of adaptability when providing implementation support [64–66]; numerous approaches to such adaptation have been described in recent years [67, 68]. Similarly, we were unable to adapt the CVD bundle tools to address user feedback; doing so might have facilitated tool adoption. Thus, the study clinics were asked to adopt imperfect tools; even minor flaws in such tools can hamper their adoption [69–72].

In addition, the CDS tools used here were far more complex than those tested in our prior study. This was driven by the need to incorporate complicated new statin guidelines into the tools, to address shortcomings of the earlier tools by incorporating them into a suite of tools targeting a broader set of care guidelines, and to work pragmatically within the decision-making structure of the EHR provider, with the attendant benefits and constraints. Though pragmatic, these changes complicated our ability to assess the impact of the implementation strategies of interest. The study CHCs were also expected to use these tools to track their own progress, but the tools proved difficult to use and had limited ability to enable retrospective data review. Such challenges to self-monitoring progress may decrease as EHRs grow increasingly user-friendly. Compatibility, complexity, and effectiveness of innovations—none of which were optimal here—are known to impact adoption decisions [73].

In our preceding study, significant impacts were seen, but the study clinics received support far more intensive than that provided to this study’s arm 3 clinics, including coaching provided by a trusted colleague [7]. It is possible that the strategies provided here were not adequate to support change in these clinics. Further research is needed to determine whether there are thresholds of support necessary, perhaps based on specific baseline characteristics of CHCs that might serve as barriers and assets to capitalizing on such support.

### Overall improvements in statin prescribing

The factors described above help explain why no additive effect of increasingly intensive implementation support was seen. Different factors drove the overall improvements seen in statin-related outcomes. Notably, these occurred concurrent with a secular trend toward guideline-concordant statin prescribing, as indicated by the comparison CHCs’ improvement. Selection bias is also possible, as those clinics who volunteered for the study may have been especially motivated to improve CVD care. Participating in the study may have focused clinic staff attention on the targeted outcomes: for example, several point people said that the regular qualitative team check-in calls maintained their focus on the improvement objectives. This increased awareness may have had an impact at sites that were already highly motivated, and only needed a small push to improve. It is also possible that our randomization process did not result in a random distribution of important factors that impacted the study CHCs’ ability/motivation to capitalize on the support they received. Notably, many of the arm 2 CHCs’ clinician champions were particularly motivated compared with those in the other arms’ CHCs. Analyses exploring the association between clinic-level factors and the study outcomes are underway and will be reported in the future.

Given that this study’s results did not support our primary hypothesis, and that differences in cost were not a primary study outcome, we chose not to conduct analyses to analyze the difference in costs of the implementation strategies compared here, although these costs certainly rose with the intensity of the provided support. If future research shows differences in impact between levels of implementation support, the costs associated with such support should be assessed.

This study’s findings are likely to have relevance for many providers of primary care but may be less generalizable to those that are in better-resourced settings and/or serving patients who are less socioeconomically vulnerable. Ideally, researchers replicating this study will conduct it within a group of clinics that share a given EHR and its decision support tools.

## Conclusion

We sought to assess whether increasingly intensive implementation support led to increasing improvement in guideline-concordant care in CHCs. Our findings did not support this hypothesis. These results have implications for future efforts to support the implementation of guideline-driven care and adoption of EHR-based decision support tools that target such care. Notably, it is important to take an adaptive approach to providing implementation support, thoroughly pilot-test any decision support tools when implementation support targets the adoption of such tools, and consider whether study participation and engagement with the study team is influencing this adoption. These results also show that just as there are barriers to implementing interventions with fidelity, there are barriers to providing implementation support as planned. Finally, these findings underscore the need for far more research on how to support implementation of innovations effectively, especially in under-resourced care settings like CHCs.

## Data Availability

The data generated during the current study will not be made publicly available but are available from the corresponding author on reasonable request

## Abbreviations

CHC: Community health center
EHR: Electronic heath record
CDS: Clinical decision support
DM: Diabetes mellitus
SPREAD-NET: Study of Practices Enabling Implementation and Adaptation in the Safety Net
ACE: Angiotensin-converting enzyme
ARB: Angiotensin-II receptor blockers
CVD: Cardiovascular disease
SQL: Structured query language
DiD: Difference-in-differences
GEE: Generalized estimating equation
RR: Rate ratio
CI: Confidence interval
FPL: Federal poverty level
PCP: Primary care provider
CFIR: Consolidated Framework for Implementation Research
PF: Practice facilitator
